# Anti-trichomonad activities of different compounds from foods, marine products, and medicinal plants: a review

**DOI:** 10.1186/s12906-020-03061-9

**Published:** 2020-09-09

**Authors:** Mendel Friedman, Christina C. Tam, Luisa W. Cheng, Kirkwood M. Land

**Affiliations:** 1 United States Department of Agriculture, Healthy Processed Foods Research Unit, Agricultural Research Service, Albany, CA 94710 USA; 2 United States Department of Agriculture, Foodborne Toxins Detection and Prevention Research Unit, Agricultural Research Service, Albany, California 94710 USA; 3 Department of Biological Sciences, University of the Pacific, Stockton, CA 95211 USA

**Keywords:** *Trichomonas vaginalis*, *Tritrichomonas foetus*, Trichomoniasis, Trichomonosis, Rodent and human studies, Food compounds, Marine compounds, Medicinal plant compounds, Anti-trichomonad effects, Mechanisms

## Abstract

Human trichomoniasis, caused by the pathogenic parasitic protozoan *Trichomonas vaginalis*, is the most common non-viral sexually transmitted disease that contributes to reproductive morbidity in affected women and possibly to prostate cancer in men. *Tritrichomonas foetus* strains cause the disease trichomoniasis in farm animals (cattle, bulls, pigs) and diarrhea in domestic animals (cats and dogs). Because some *T. vaginalis* strains have become resistant to the widely used drug metronidazole, there is a need to develop alternative treatments, based on safe natural products that have the potential to replace and/or enhance the activity of lower doses of metronidazole. To help meet this need, this overview collates and interprets worldwide reported studies on the efficacy of structurally different classes of food, marine, and medicinal plant extracts and some of their bioactive pure compounds against *T. vaginalis* and *T. foetus* in vitro and in infected mice and women. Active food extracts include potato peels and their glycoalkaloids α-chaconine and α-solanine, caffeic and chlorogenic acids, and quercetin; the tomato glycoalkaloid α-tomatine; theaflavin-rich black tea extracts and bioactive theaflavins; plant essential oils and their compounds (+)-α-bisabolol and eugenol; the grape skin compound resveratrol; the kidney bean lectin, marine extracts from algae, seaweeds, and fungi and compounds that are derived from fungi; medicinal extracts and about 30 isolated pure compounds. Also covered are the inactivation of drug-resistant *T. vaginalis* and *T. foetus* strains by sensitized light; anti-trichomonad effects in mice and women; beneficial effects of probiotics in women; and mechanisms that govern cell death. The summarized findings will hopefully stimulate additional research, including molecular-mechanism-guided inactivations and human clinical studies, that will help ameliorate adverse effects of pathogenic protozoa.

## Background

The flagellate protozoan *Trichomonas vaginalis* is an extracellular parasite that infects the vagina and the male genital tract causing the most common non-viral sexually transmitted human venereal disease (STD) trichomoniasis, with about 300 million annual cases worldwide and about 3.7 million in the United States [1–3].

The mostly asymptomatic disease does not seem to decrease with age, reaching a maximum rate in 48–51-year old women [4]. Metronidazole (5-nitroimidazole) seems to be the one of the few available synthetic drugs used clinically to treat trichomoniasis. Coinfection with other STDs is common; chlamydia, gonorrhea, and syphilis are other STDs that cause major health problems, especially in tropical and subtropical developing countries [5]. *Tritrichomonas foetus* strains cause trichomonosis, STDs in cattle [6], and pigs [7], and diarrhea in cats and dogs [8–10].

Because these pathogens are developing resistance to the widely used drug metronidazole and the disease causes numerous unpleasant adverse side effects (allergy, nausea, vomiting, predisposition to cervical cancer, adverse pregnancy outcomes, infertility, and a co-factor in the transmission of the human immunodeficiency virus (HIV), there is an urgent need to develop new effective therapeutic agents against both drug sensitive and resistant trichomonads that might be able to protect against and/or help cure exposed individuals and animals. To help meet this need, this review collates and interprets studies from several countries on reported anti-trichomonad properties of a variety of structurally different food, marine, and medicinal, including herbal, plant compounds, as well marine, plant, and fungal extracts containing multiple bioactive compounds. The results of the described efforts suggest that several formulations (pure compounds and extracts) could replace or enhance additively and synergistically the therapeutic potency of metronidazole of 30 anti-trichomonad compounds shown in Table 1 and described in the text.

**Table 1:** Inhibition of pathogenic trichomonads by food and medicinal plant compounds listed alphabetically

Compound	Source	Trichomonad	Inhibition	Reference
Benzopyrans	medicinal plant; *Hypericum polyanthenum*	*T. vaginalis*	cell damage	[11]
Betulinic acid	medicinal plant; *Palatanus acerifoli*	*T. vaginalis*	cell growth	[12]
(+)-Bisabolol	essential oil; *Nectandra megapotamica*	*T. vaginalis*	IC_50_ 98.7 μg/mL	[13]
Caffeic acid	potatoes; *Solanum tuberosum*	3 trichomonads ^a^	21.1–42.8%	[14]
Candimine	ornamental plant; *Hippeastrum morelianum*	*T. vaginalis*	cell damage	[15]
Carmaphycin-17	cancer drug; protesome inhibitor	*T. vaginalis*	highly active	[16]
α-Chaconine	potatoes; *Solanum tuberosum*	3 trichomonads	IC_50_ 35-60 μM	[14]
Chlorogenic acid	potatoes**;** *Solanum tuberosum*	3 trichomonads	11.4–21.9%	[14]
Emodin	rhubarb; *Rheum palmatum*	*T. vaginalis*	active in mice	[17]
Geraniol	essential oil; *Amomum tsao-ko*	*T. vaginalis*	171-343 μg/mL	[18]
Hedargenin	medicinal plant; *Cassnia holstii*	*T. vaginalis*	IC_50_ 2.8 μM	[19]
(+)-Isoaustrobrasilol	medicinal plant; *Hypericum* spp.	*T. vaginalis*	cell damage	[20]
Lectin	kidney beans; *Phaseolus vulgaris*	*T. vaginalis*	cell damage	[21]
Lucidin-isopropyl-ether	plant roots; *Morinda panamensis*	*T. vaginalis*	IC_50_ 1.32 μg/mL	[22]
Lycorine	ornamental plant; *Hippeastrum breviflorum*	*T. vaginalis*	cell damage	[23]
Lycosinine	ornamental plant; *Hippeastrum breviflorum*	*T. vaginalis*	cell damage	[23]
Methyl jasmonate	plant hormone	*T. vaginalis*	cell death	[24]
N-acetyl-L-cysteine	L-cysteine *amino acid*	*T. vaginalis*	active in vivo	[16]
Pyrrolocin A	fungal endophyte E6927E	*T. vaginalis*	EC_50_ 60 nM	[25]
Quercetin	potatoes; *Solanum tuberosum*	3 trichomonads	18.5–46.6%	[14]
Resveratrol	grapes; *Vitis vinifera*	*T. vaginalis*	IC_50_ 25 μM	[26]
Saponins A, B	medicinal plant; *Sapindus saponaria*	*T. vaginalis*	MIC 0.025%; MIC 0.16 mg/mL	[27] [28]
Solanidine	potatoes; *Solanum tuberosum*	3 trichomonads	22.6–48.4%	[14]
α-Solanine	potatoes; *Solanum tuberosum*	3 trichomonads	IC_50_ 10.9–16.8 μM	[14]
Tomatidine	tomatoes; *Lycopersicon esculentum*	3 trichomonads	3.2–22.9%	[29]
Tomatine	tomatoes; *Lycopersicon esculentum*	3 trichomonads	IC_50_ 2.0–7.9 μM	[29]
Torvosides	medicinal plant; *Solanum torvum*	*T. vaginalis*	MIC 6.2–12.5 μM	[30]
Uliginosin B	medicinal plant; *Hypericum polyanthenum*	*T. vaginalis*	cell damage	[11]
Ursolic acid	medicinal plant; *Manika rufula*	*T. vaginalis*	MIC 25 μM	[31]
Wogonine	plant leaves; *Scutellaria havanensis*	*T. vaginalis*	cytotoxicity	[32]

^a^Bovine, feline, and human trichomonad strains

### Vaginal microflora, dysbiosis, disease, and treatment

Because vaginal dysbiosis is a contributing factor to trichomoniasis in women and because treatments applied vaginally should not upset this important ecosystem, it is worth considering the effects of this dysbiosis and how balance can potentially be restored with biotic treatments. The role of the “normal” microflora has been extensively studied, especially in relation to human gastrointestinal diseases and susceptibility to pathogens and toxins. The dysbiotic microflora environment can be reversed via treatment with prebiotics and/or probiotics such as lactobacilli with beneficial results for a variety of gastrointestinal diseases and reduced susceptibility to pathogens and pathogen products [33]. Dysbiosis or disturbance of this very important microflora in the vagina can lead to many severe adverse conditions including preterm birth, pelvic inflammatory disease, increased risk and transmission of sexually transmitted infections, along with many other health conditions [34]. Therefore, understanding the effects of the vaginal microbiome and its dysregulation on human health and susceptibility to trichomonad infection is of crucial importance. Additionally, researchers must understand the urogenital, and specifically the vaginal environment to safely and effectively deliver therapeutics to target the infection.

A normal vagina is acidic with a pH of approximately 4 to 4.5 but can vary between 3.5 and 5. This acidic environment occurs because of the resident microflora. It has been reported that there are five predominant microbiota states or community types (CSTs) that have been isolated from premenopausal women. Four of these CSTs are dominated by *Lactobacillu*s spp. The last CST (CST-IV), however, does not contain lactobacilli but rather the overgrowth of anaerobic bacteria i.e. *Gardnerella vaginalis*, *Atopobium vaginae*, *Prevotella bivia*, and others [35–37].

The lactobacilli use glycogen from exfoliated epithelial cells, which is converted to lactic and fatty acids. Additionally, these bacteria are able to produce other bactericidal products including hydrogen peroxide, bacteriocins, bacteriocin-like products, and surfactants that help in preventing infections [38, 39]. Products from various *Lactobacillus* spp. have been shown to inhibit or reduce adherence of urogenital pathogens via competitive exclusion of the host receptor(s) [38]. Phukan, Brooks [40] described the discovery of an aggregation promoting factor APF-2 from the vaginal strain *L. gasseri* ATCC 9857 that inhibits *T. vaginalis* adherence to human vaginal ectocervical cells. Hinderfeld, Phukan [37] showed that *T. vaginalis* and CST-IV associated-bacteria are correlated with *T. vaginalis* infections [41], can enhance paracellular permeability of the cervicovaginal epithelium by dysregulating tight junctions, and affect proinflammatory cytokines such as interleukin-6 (IL-6) and tumor necrosis factor-α (TNF-α). Valadkhani et al. indicated the protective role of *Lactobacillus acidophilus* in *T. vaginalis* infection using healthy human vaginal cells [42]. Restoration of the vaginal microflora by both oral and vaginal delivery of prebiotics, probiotics, and/or synbiotics have been attempted for bacterial vaginosis, as well as for *T. vaginalis* infection, with varying degrees of success dependent on strain(s) used, duration of treatment, route of delivery, and whether in combination with traditional drugs [39, 43]. This is therefore a potential strategy that could be investigated alongside the natural products that we will describe herein.

### Anti-trichomonad compounds in plant-based food

Plants produce bioactive organic compounds known as secondary metabolites to protect themselves against various diseases caused by phytopathogens as well as by environmental stress conditions such as drought. Some of these compounds are therefore found in plant-based foods and it is known that they also have the potential to protect animals and humans against different pathogenic organisms, including parasitic protozoa.

### Potato glycoalkaloids – α-chaconine, α-solanine, and the aglycone solanidine

**Fig. 1:**
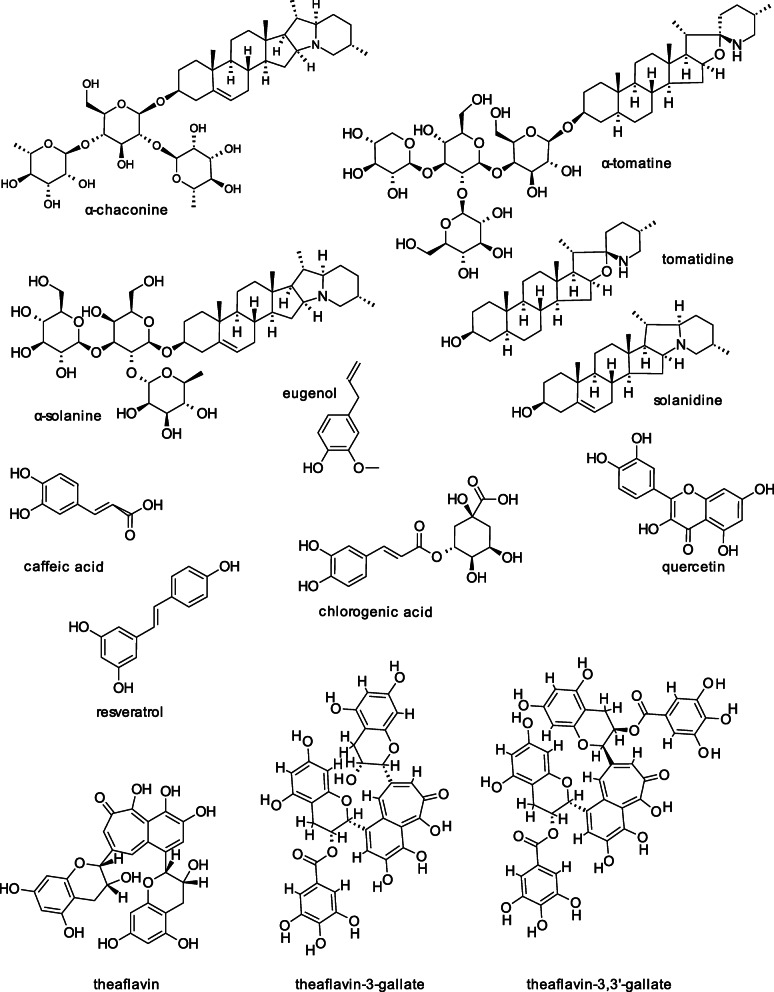
Structures of anti-trichomonad food compounds

Friedman, Huang [14] describe the inactivation, using cell-based assays, of the three pathogenic protozoal strains *T. vaginalis* human G3, *Tritrichomonas foetus* feline C1, and *Tritrichomonas foetus* bovine D1 by the potato (*Solanum tuberosum*) glycoalkaloids α-chaconine and α-solanine, their common aglycone solanidine, three potato phenolic compounds (Fig. 1), and six potato peel powders. The two glycoalkaloids completely inhibited all three strains under the test conditions, with the following IC_50_ values (concentration that inhibited 50% of the cells under the test conditions) calculated from concentration-response data: for G3, α-solanine 15.8 μM and α-chaconine 35.6 μM; for C1, α-solanine 12.6 μM and α-chaconine 51.5 μM, and for D1 α-solanine 10.9 μM and α-chaconine 35–60 μM. Solanidine was less active, inactivating G3, C1 and D1 to 48.4, 22.6, and 23.0%, respectively. IC_50_ values were not calculated for inhibitory activities of < 50%. Three potato phenolic compounds also inactivated the three strains. Caffeic acid inhibited G3 to 42.8%; D1 to 43.7%; and C1 to 21.1%. The corresponding values for chlorogenic acid are 11.4, 12.1, and 21.9%, and for quercetin 45.6, 18.9, and 18.5%, respectively. Additional studies found that six potato peel powders containing glycoalkaloids and other bioactive compounds [44] also inhibited the three trichomonads. The percentage inhibition of G3 ranged from 1 (red potato peel) to 36.6 (organic Russet potato peel); of D1 from 9.0 (red potato peel) to 41.4 (organic Russet potato peel); and of C1 from 0 (purple potato peel) to 48.6 (organic Russet peel). Disc-diffusion screening revealed no effect on normal microbiota in the vaginal flora.

The cited data indicate that the activities of the potato glycoalkaloids are greater than that of their common aglycone, that the activities of the phenolic compounds are also lower than those of the glycoalkaloids, and that, of the six evaluated peels, the organic Russet potato peels showed the greatest anti-trichomonad activity. These positive results imply that the test formulations might be able to protect both humans and animals from trichomonad infections. Moreover, in previous studies we reported that the two potato glycoalkaloids inhibited the growth of cancer cells [45, 46], and that, compared with the other peel powders, the organic Russet potato peel powder induced the greatest weight loss in mice on a high-fat diet [47], suggesting that the potato glycoalkaloids have multifunctional health properties. Will inexpensive Russet potato peel powders protect humans, farm animals, and cats against cancer, obesity, and trichomoniasis? Will the consumption of baked and boiled potatoes with the peels benefit human health? We are challenged to find answers to these questions.

### The tomato glycoalkaloid α-tomatine and the aglycone tomatidine

Liu, Kanetake [29] reported the following experimental observations on the inactivation of three pathogenic protozoal strains (G3, human *T. vaginalis*; D1, cattle *T. foetus*; and C1*,* feline *T. foetus*) by the commercial tetrasaccharide tomato glycoalkaloid tomatine (a 9:1 mixture of α-tomatine and dehydrotomatine from tomatoes (*Lycopersicon esculentum*, Fig. 1) and the aglycone tomatidine lacking the carbohydrate side chain. Under the test conditions, tomatine completely inactivated the three strains, whereas the corresponding inactivation by tomatidine was much lower for all three strains (3.2, 22.9, and 10.2%, for G3, D1, and C1, respectively). The calculated IC_*50*_ values for tomatine based on dose-response data of G3, D1, and C1 are 7.9 μM, 2.7 μM, and 2.0 μM, respectively, which compares with IC_50_ values for metronidazole of 0.72 μM, 0.49 μM, and 0.55 μM, respectively (the much lower values indicating inhibition is greater). The results show that the feline trichomonad strain was the most susceptible to inactivation by tomatine. Unlike medicinal antibiotics, tomatine did not affect normal flora bacteria in vitro.

Because the aglycone tomatidine showed lower activity than the glycoside tomatine, it seems likely that the tetrasaccharide side chain is involved in the molecular mechanism of inactivation of the trichomonad cells, possibly by binding to and disrupting the cell membranes, as described by Blankemeyer et al. [48, 49] and de Groot and Müller-Goymann [50]. It would be of interest to find out if inexpensive extracts of green tomatoes and tomato leaves with a high-tomatine content [51, 52] would also be effective against the human, bovine, and feline trichomonads.

Tomatine has also been reported to have other health-promoting activities, such as reducing plasma cholesterol levels in hamsters [53], and inhibiting the growth of cancer tumors in fish [54], and in mice [55], making it a good target for further investigation in human studies. Interestingly, tomatine content decreases as the fruit matures on the vine so green, unripe tomatoes can have about 100 times more tomatine than mature red [56], and tomatine is found in processed tomato products including sauce, ketchup, and pickled tomatoes [57]. Jars of high-tomatine pickled green tomatoes are available in some stores.

In related studies Chen, Li [58] showed that five glycoalkaloids, α-chaconine, α-tomatine, α-solanine, α-solamargine, and α-solasonine, inhibited the growth of the malaria-causing protozoan *Plasmodium yoelii* in a 4-day study in mice, suggesting that the last two compounds also merit evaluation against *T. vaginalis* and *T. foetus.* Thorne, Clarke [59] reported that α-chaconine, α-tomatine, and α-solasonine inactivated the herpes simplex virus Type I in cell culture, suggesting that these glycoalkaloids might show anti-viral properties against the coronavirus, human immunodeficiency (HIV), and other pathogenic viruses.

### Lectin from kidney bean

Aminou, Alam-Eldin [21] also reported that the biologically active glycoprotein, a lectin from the kidney bean (*Phaseolus vulgaris*) known to agglutinate (precipitate) cells, induced structural changes in the *T. vaginalis* structure, suggesting that this and other lectins have the potential to act as anti-trichomonad agents. The lectin was also used as a probe against cerebral spinal fluid from patients in an enzyme-linked lectin assay (ELLA) to determine their risk of developing Alzheimer disease [60].

### Phytochemical-rich food extracts

Beverages and foods rich in phytochemicals have also been shown to have inhibitory activity towards trichomonads. For example, using a cell assay, Noritake, Liu [61] determined the inhibitory activities of black tea, green tea, grape, pomegranate, and jujube extracts and dried jujube against *T. vaginalis* G3, *T. foetus* D1, and *T. foetus* C1. The results show that the black tea extract inactivated the three trichomonads and was most effective against *T. vaginalis*. The extract was also effective against a metronidazole-resistant strain and a cytoadherent strain of *T. vaginalis.* The inhibition correlated with the total extract and individual component [(theaflavin, theaflavin 3-gallate, and theaflavin-3,3′-digallate (Fig. 1)] content of the extracts as determined using HPLC. The black tea extract did not affect normal flora bacteria in vitro.

The inactivation by the catechin-containing green tea and other extracts was variable and lower than that by the black tea extracts. The authors suggest that black tea and its bioactive theaflavin compounds merit further human clinical studies with *T. vaginalis*. Related studies show that individual green tea catechins and black theaflavins and tea extracts inhibited the growth of pathogenic bacteria and viruses [62, 63], and multiple human cancer cells [64], and that the theaflavin and catechin content of 77 commercial green and black teas varied widely [65, 66], suggesting that clinicians and tea consumers should select teas with the highest content of the bioactive compounds.

Three publications by Sirk et al. [67–69] describe studies on molecular-dynamics computer simulations of green tea catechins and black tea theaflavins and lipid bilayers of cell membranes. The simulations show that the tea compounds have a strong affinity for the lipid bilayer; some are absorbed into the bilayer and some remain on the surface. The molecular structure of the compounds influences their absorption, as well as the ability of their hydroxyl (OH) groups to form hydrogen bonds with the lipid bilayer headgroups. Insight into these molecular events facilitates a better understanding of the mechanism of disruption of cell membranes in biological processes, including the anti-cancer, antibacterial, and antiprotozoal effects.

### Plant essential oils and their bioactive compounds

**Fig. 2:**
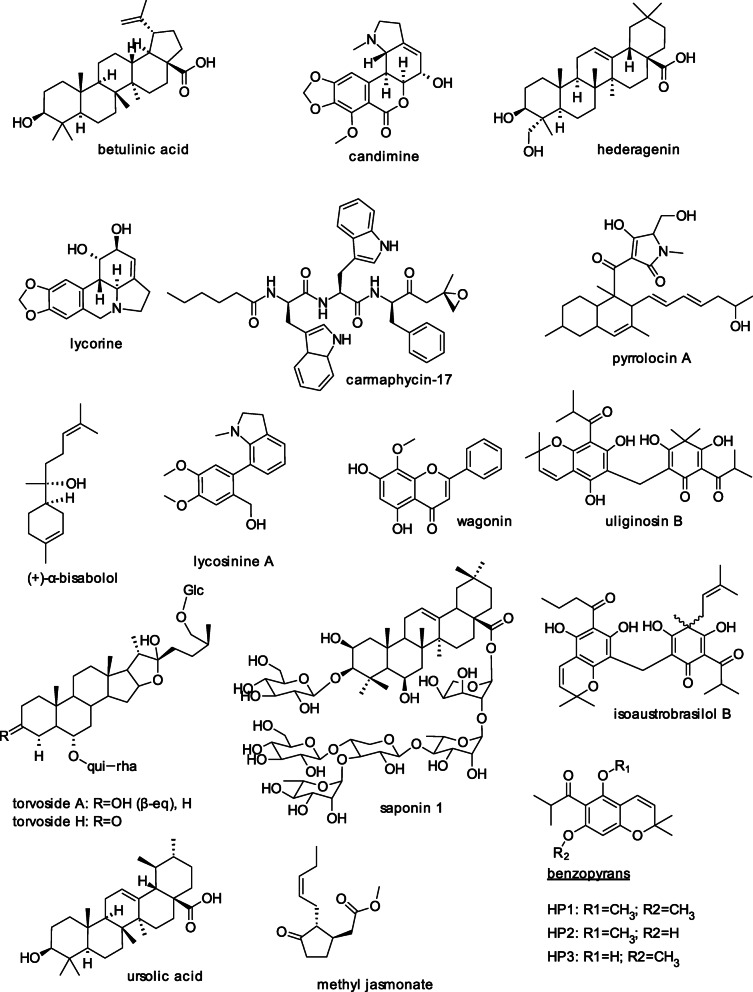
Structures of anti-trichomonad medicinal plant and marine compounds

There have been reports on the anti-trichomonad properties of plant essential oils and compounds isolated from the oils, as illustrated with the following examples. An Australian study by Moon, Wilkinson [70] describes the inhibition of three protozoan pathogens by *Lavandula* essential oils. Concentrations of < 1% of *L. angustifolia* and *L. × intermedia* oils completely eliminated the human pathogens *T. vaginalis* and *Giardia duodenalis* and the fish pathogen *Hex amita inflata*. Cheikh-Ali, Adiko [71] reported that the essential oil from rhizomes of *Aframomum sceptrum* (Zingiberaceace) exhibited remarkable antiprotozoal activity against *T. vaginalis* with an IC_50_ value of 0.12 μL/mL and a minimum lethal concentration (MLC) value of 1.72 μL/mL. The major constituents of the oil determined by gas chromatography/mass spectrometry (GC/MS) analysis are β-pinene (12.7%), caryophyllene oxide (10.0%), and cyperene (6.0%). The oil also inhibited the growth of Gram-positive bacteria. Dai, Peng [18] found that *A. tsao-ko* essential oil used in traditional Chinese medicine to treat stomach disorders and throat infections and its bioactive compound geraniol (Fig. 2), widely used as a fragrance ingredient, were also effective against *T. vaginalis*. The MLC of the oil for one strain of the pathogen was 44.97 μg/mL and the IC_50_ value, 22.49 μg/mL. These values were approximately double with a second protozoal strain. The corresponding value for geraniol, which constitutes 13.69% of the oil, are 342.96 μg/mL and 171.48 μg/mL, respectively. These results show that geraniol showed much lower potency than the oil, suggesting that, in addition to geraniol, some of the 24 compounds identified in the oil by GC-MS must contribute to its potency. Additional studies showed that the oil induced severe morphological changes in the cells. These included partially damaged cytoplasmic membranes, resulting in leakage of cytoplasmic content that presumably led to cell death. Aminou, Alam-Eldin [21] discovered that the oil from the seeds of the *Nigella sativa* plant (black seed, black cumin spice) native to Egypt induced cell damage with cytoplasmic and nuclear destruction in the ultrastructure of *T. vaginalis* trophozoites, detected by transmission electron microscopy, that is similar to the effect induced by metronidazole, suggesting that the oil might be a useful therapeutic alternative to the synthetic drug. The alcoholic extract was less active than the oil. The authors speculate that the anti-trichomonad effect might be associated with high content of poly-unsaturated linoleic, oleic, and palmitic acids in the oil. Akram Khan and Afzal [72] reported that *Nigella sativa* seeds contain bioactive alkaloids. Another study by Shaikh, Aaqil [73] describes the use of a molecular docking method that demonstrated that extracts of plant *Apamarga Kshara*, known to ameliorate cervical erosion in humans, interacted with the amino acid residues of the *T. vaginalis* enzyme carbamate kinase, suggesting that the enzyme might present a biological marker for the inhibition of the trichomoniasis.

Farias, Kato [13] found that essential oils from different *Nectandra* plant species grown in Brazil contained a variety of compounds, including the phenylpropanoid derivative (+)-α-bisabolol (Fig. 2). The oil isolated from the leaves of *Nectandra megapotamica* contained 93.7% (+)-α-bisabolol. This compound inhibited biofilm formation in methicillin-resistant *Staphylococcus aureus* and *Pseudomonas aeruginosa* bacteria and was also active against *T. vaginalis* with an IC_50_ value of 98.7 μg/mL. It also had cytotoxic and hemolytic effects in Vero cells and human erythrocytes. Oliveira, Freitas [74] found that (+)-α-bisabolol acted synergistically with the antibiotic drug gentamicin against *Escherichia coli*. These results suggest the potential value of *Nectandra* oils and their bioactive compounds in the treatment of infectious diseases caused by clinical and foodborne pathogenic bacteria and by *T. vaginalis*, as well its potential to protect foods against contamination by both microbial and protozoal pathogens.

The fact that essential oils and oil compounds inactivated both pathogenic bacteria and *T. vaginalis* suggest that oils/oil compounds that have been shown to inactivate foodborne microorganisms in vitro and in contaminated food might also be effective against *T. vaginalis*. For example, we reported that 27 of the evaluated 96 essential oils and 12 of the 23 essential oil compounds inactivated multiple species of foodborne pathogenic bacteria (*Campylobacter jejuni*, *E. coli*, *Listeria monocytogenes*, and *Salmonella enterica*) in vitro [75, 76]. Essential oils and oil compounds also inactivated pathogenic bacteria in apple juice [77], ground pork [78], and on leafy greens [79], suggesting that some of the commercially available, food-compatible essential oils/oil compounds might also inhibit the growth of pathogenic protozoa. Will safe, food-compatible oils/oil compounds (e.g. allspice, cinnamon, clove, lemongrass, oregano, and thyme essential oils) and their bioactive compounds (e.g. carvacrol, cinnamaldehyde, citral, eugenol, geraniol, thymol) added to liquid and solid foods protect consumers against both microbial infections and trichomoniasis?

### Manuka honey

A study by Sinha, Prakash [80] reported that manuka honey derived from the *manuka sexta* bees feeding from the manuka tree (*Leptospermum scoparium*) [81], is known to have anti-microbial and wound-healing properties, inhibited the growth of the pathogenic protozoa *Giardia lamblia* and *T. vaginalis* with IC_50_ values of 5.6%v/v and 1.5%v/v, respectively, suggesting that the honey has the potential to ameliorate human giardiasis and trichomoniasis. We do not know the bioactive components of the honey that might be responsible for these beneficial properties.

### Resveratrol from grapes

Mallo, Lamas [26] analyzed the in vitro effects of the natural polyphenolic compound resveratrol (Fig. 1) present in grape skins [82], against *T. vaginalis*. The effect was cytostatic at a concentration of 25 μM and cytotoxic at 100 μM, with calculated mean IC_50_ values of 32 μM on day 1 and 25 μM on day 2, respectively. The corresponding IC_50_ values using metronidazole are 3.2 μM and 4.25 μM, respectively. Although the kinetics of the in vitro inhibition of the *T. vaginalis* cells by resveratrol and metronidazole are similar, in terms of IC_50_ values, metronidazole was about 6- to 10-fold more effective than resveratrol.

The authors also obtained cellular and molecular evidence for the possible mechanism of action of resveratrol against the parasite, as indicated by the following findings. Resveratrol acted primarily as an inhibitor of the enzyme 120-kDA [Fe] hydrogenase (Tvhyd), upregulated the enzyme hydrogenosomal pyruvate-ferrodoxin oxidoreductase, and induced overexpression of the heat-shock protein 70 (Hsp 70), a protective protein found in the hydrogenosome of *T. vaginalis*. The authors suggest that the deleterious inhibitory effect of resveratrol resulted from the inhibition of hydrogen production in the hydrogenosomes and changes in iron transport, causing a high degree of bioenergetic stress in the treated parasites. This mechanism differs from the antioxidative mechanisms that have been proposed for other multi-beneficial effects of resveratrol, including cancer [83] and heart disease [84]. Additional studies by several investigators designed to elucidate mechanistic aspect of anti-trichomonad effect in *T. vaginalis* are highlighted below in a separate Mechanisms section.

### Marine compounds

There have been a few limited studies on effects of marine extracts and isolated compounds on *T. vaginalis*. A screen by Moo-Puc, Robledo [85] of 25 tropical seaweeds from the Yucatan coast of Mexico showed that 44% of the extracts had high to moderate activity against *T. vaginalis*, with *Lobophora variegata* and *Udotea conglutinata* species showing the maximal activity with IC_50_ values of 1.39 and 1.66 μg/mL, respectively. Cantillo-Ciau, Moo-Puc [86] reported that an extract from the alga *Lobophora variegata* from the Yucatan coast of Mexico showed high activity against *T. vaginalis* with an IC_50_ value of 3.2 μg/mL. Several isolated and chemically characterized compounds were less active with an IC_50_ value of 8.0 μg/mL. Scopel, dos Santos [87] found that two marine-associated fungal species (*Hypocrea lixii* FO2 and *Penicillium citrinum* F40) isolated from 39 different marine organisms, mainly sponges, from the South Brazilian coast showed up to 100% growth-inhibitory activity against *T. vaginalis* fresh, clinical, and a metronidazole-resistant isolates at a concentration of 2.5 mg/mL. The negative results of a hemolytic assay show that both samples were compatible with red blood cells, suggesting that these samples are potential candidates against *T. vaginalis*. The cited studies suggest that the sea algae, seaweed, fungal and other species could serve as a source of highly active anti-trichomonad formulations.

### Medicinal plants: extracts and bioactive compounds

#### Plant extracts and bioactive compounds – in vitro studies

Here, we briefly outline results from selected reported studies on anti-*T. vaginalis* properties of medicinal plant extracts.

Muelas-Serrano, Nogal [88] reported that, of a large number evaluated American plant extracts, nine were active against *Trypanosoma cruzi* and *Trichomonas vaginalis*, with one showing 100% inhibition of *T. vaginalis* after exposure for 24 h. A screen by Calzada, Yépez-Mulia [89] of 22 Mexican medicinal plants for anti-trichomonal activity against *T. vaginalis* indicated that extracts from *Garica papaya* and *Cocos nucifera* had the best activity, with IC_50_ values of 5.6 and 5.8 *μ*g/mL, respectively. All extracts, however, were less active than metronidazole, with an IC_50_ value of 0.037 *μ*g/mL. Five of the evaluated plants are used to treat urogenital tract disorders in Mexican traditional medicine.

Arthan, Sithiprom [30] investigated the effectiveness of compounds isolated from plants grown in Thailand against *T. vaginalis*. The MICs of several β-glycosides at 24 h were in the range of 6.25–12.5 *μ*M. The glycosides torvosides A and H (Fig. 2) were more potent than their corresponding deglucosylated torvoside A and H, whereas other β-glycosides were generally as active as their corresponding aglycones. Except for dalcochinin, none of the tested compounds showed cytotoxicity against Vero and cancer cell lines (KB and MCF-7). The native Thai plants provide another natural source of anti-*T. vaginalis* compounds.

Fernández-Calienes Valdés, Monzote Fidalgo [32] found that extracts of leaves and stems of *Scutellaria havanensis*, a plant native to Cuba, and the isolated flavonoid compound wogonin (Fig. 2) exhibited anti-trichomonad activity against *T. vaginalis* with the following selectivity indices: methanol extract 7.3; chloroform extract 2.8; wogonin 2.0; and metronidazole 322.6. Because antiprotozoal efficacy must have a selectivity index of at least 10, defined as the ratio of concentrations that measures cytotoxic to antiprotozoal activity [90], the authors suggest that the observed activities are probably due to unspecific cytotoxic rather than specific anti-trichomonad effects. The test substance also exhibited activity in vitro against *Plasmodium berghei* parasites, suggesting their possible value against malaria.

Menezes, Rigo [20] evaluated the mechanism of the anti-trichomonad effect of phloroglucinol compounds isolated from a Brazilian *Hypericum* plant species. The compound isoaustrobrasilol B (Fig. 2) significantly inhibited nucleoside triphosphate diphosphohydrolase (NDPDase) and ecto-5’nucleotidase activities as well as immune changes attributed to extracellular nucleotide accumulation, suggesting that the death of *T. vaginalis* cells and modulation of electonucleotidases induced by the phloroglucinol derivative may increase the susceptibility of the parasite to host immune cells (neutrophils), thus enhancing cell death. A review of the literature of 26 plant types by Ziaei Hezarjaribi, Nadeali [91] indicates that extracts of medicinal herbs such as *Artemisia*, *Zataria multiflora*, and *Lavandula angustifolia* are remarkably effective against *T. vaginalis*, with the most effective *Artemisia aucheri* extract active at a concentration of 0.1 mg/mL.

Because the activities of plant extracts might be associated with a mixture of bioactive compounds that might act additively, synergistically, or antagonistically, there is a need to isolate and determine activities of the pure compounds, as indicated below for some extracts.

#### Plant extracts - human studies

The following studies demonstrate the effectiveness of medicinal plants in alleviating trichomoniasis in women and another study shows the potential of microbiota to increase the therapeutic potential of metronidazole. A randomized study by Moraes, Cunha [92] of the treatment of 60 Brazilian women infected with *T. vaginalis* with 24 mg of the herbal plant *Mentha crispa* (Lamiaceae family) or 2000 mg of secnidazole (5-nitroimidazole, structurally related to metronidazole) administered orally in tablet form, found that: (a) there was no statistical difference between the two treatments in the observed cure rates, 90 and 96.6% (*p* = 0.621), respectively; (b) the adverse effects (mostly nausea, metallic taste) were significantly higher (*p* = 0.05) in the secnidazole (66.66%) than in the *M. crispa* group (20%), *p* = 001). The cited results show that *M. crispa* seems to offer an alternative and safe treatment for trichomoniasis, and possibly also for giardiasis [93]. It is, therefore, surprising that *M. crispa* has not been adopted for wider clinical evaluation and use in view of its striking beneficial effect at a much lower dose than the synthetic drug.

Abdali, Jahed [94] used a randomized study of 420 women of reproductive age to determine the effect of a vaginal cream containing a medicine made from *Zataria multiflora* (Labiatae family) against *T. vaginalis* vaginal infections and bacterial vaginosis that often accompanies the protozoal infection. The control group was administered oral metronidazole tablets. The results show that the cream is as effective as the drug in the treatment of bacterial vaginosis (*p* = 0.01) and clinical symptoms associated with *T. vaginalis* (*p* = 0.001). The authors suggest that the cream should also be evaluated for the possible recurrence of *T. vaginalis* following treatment. A study by Sgibnev and Kremleva [95] found that twice daily intravaginal co-administration of 500 mg metronidazole with 1 capsule of the commercial probiotic Gynophilus® to 90 women with *T*. *vaginalis* for 7 days significantly increased the therapeutic potency of the drug (88.6 and 42.9% for trichomoniasis and 63.6 and 11.9% for bacterial vaginosis (BV) treated with and without added probiotic, respectively). The binary treatment also decreased the inflammatory response and the pH and increased the redox potential in the vagina. The authors suggest that these changes are the reason for the increased therapeutic potency. The binary therapy merits further study with some of the described highly active natural anti-trichomonad compounds and extracts. A new vaginal cream containing a mixture of extracts of *Eucalyptus camaldulensis* leaves*, Viola odorata* root, and *Mentha piperita* leaf completely inhibited *T*. *vaginalis* strain G1 in vitro in the first 24 h, suggesting its potential value for human use [96].

#### Plant compounds

Here, we present highlights of reported observations on the anti-*T. vaginalis* properties of selected pure compounds isolated from medicinal plants and the use of a murine model to test anti-trichomonad properties in vivo.

### Anthraquinones

#### Emodin

**Fig. 3:**
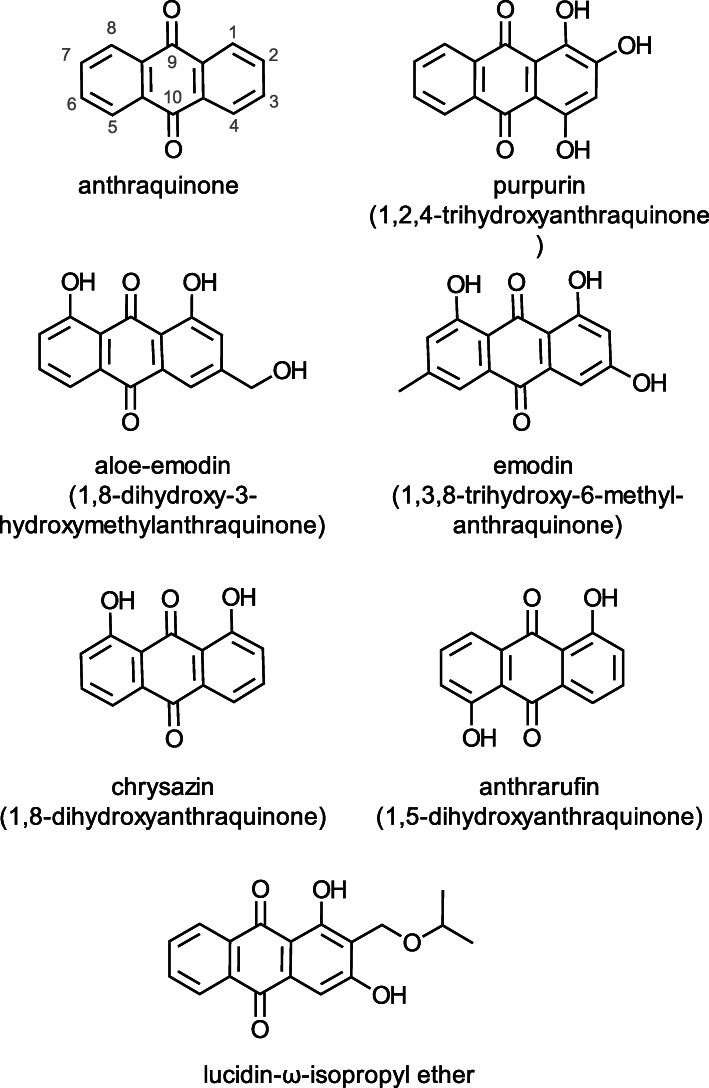
Structures of plant-derived anti-trichomonad anthraquinones

Wang [17] reported that the anthraquinone derivative emodin (1,3,8-trihydroxy-6-methyl-anthraquinone) (Fig. 3) isolated from the root and rhizome of *Rheum palmatum* inhibited the pathogenicity of *T. vaginalis* in mice. The compound delayed the development of subcutaneous abscesses due to infection by this parasite. It also cured the intravaginal infection of trichomonads through oral administration. In cell cultures, it reduced the cytotoxic effect of the parasite towards mammalian cells. This inhibition was markedly reversed by the coexistence of free radical scavengers, indicating the possible mediation of reactive oxygen species (ROS) in the pathogenicity and inhibition. Other investigators reported that emodin also inhibited breast cancer growth [97], showed antibacterial and antiviral activities when incorporated into model membranes (liposomes) as the vehicle [98], suppressed nonresistant and antibiotic-resistant pathogenic *Staphylococcus aureus* bacteria [99], and protected against oxidative damage induced by oxidized fish oil [100], suggesting that the compound has the potential to improve both food safety and human health.

Although Cobo, Eckmann [101] suggest that their murine model of vaginal trichomonad infections is a valuable approach for investigating in vivo pathogenesis and treatment of trichomoniasis, to our knowledge, the above-mentioned study of emodin is the only one that has used mice to determine the efficacy of a natural product. A murine model was also used by Hopper, Yun [102] who reported that the oral administration of the synthetic drug auranofin to mice for 4 days cleared *T. foetus* infection without any apparent side effects. Some of the described highly active natural plant extracts and pure compounds merit further evaluation in mice as a possible intermediate test that might help design and guide clinical evaluations in humans.

#### Lucidin-ω-isopropyl ether

Cáceres-Castillo, Pérez-Navarro [22] isolated and characterized the structure of a new anthraquinone (lucidin-ω-isopropyl ether) (Fig. 3) from the roots of the plant *Morinda panamensis* and determined its activity against *T. vaginalis*. The following methods were used to obtain pure test samples of the anthraquinone. The plant samples were washed under tap water and left to dry inside an oven at 40 °C for 3 days. The dry material was then ground to a 2-mm mesh diameter. The resulting powders were extracted with methylene chloride in a Soxhlet apparatus, and the concentrated residue was then fractionated on a chromatography column packed with silica gel followed by thin-layer chromatography. Nuclear magnetic resonance spectroscopy (NMR) was used to facilitate assigning the structure of the anthraquinone derivative shown in Fig. 3. The isolated anthraquinone, with an IC_50_ value of 1.32 μg/mL, was found to be similar to efficacy to the metronidazole concentration tested (6 μM = 1.03 μg/mL). The compound was also subjected to a series of additional bioassays that showed it was highly selective towards trichomonad trophozoites and was capable of inhibiting their ability to kill HeLa cells and to decrease the proteolytic activity of the proteinase TvMP50 from the trichomonad that was associated with decreased expression of the mp50 gene. The authors suggest that the high trichomonacidal activity of the new anthraquinone merits further action-mode studies to define the mechanism of action.

### Structure–activity relationships of anthraquinones

We evaluated the anti-trichomonad activities of six anthraquinones, the structures of which are shown in Fig. 3, against the three pathogenic protozoal strains G3 (human), C1 (bovine), and D1 (feline) mentioned earlier [103]. The following are the results under the test conditions in terms of percentage inactivation of G3, C1 and D1, respectively: aloe-emodin, 82.3, 99.0, and 92.0; anthraquinone, 62.0, 51.3, and 63.7; anthrarufin, 53.4, 60.0, and 73.4; chrysazin, 56.7; 91.1, and 97.4; emodin, 57.7, 41.4, and 78.8; and purpurin, 58.8, 72.2, and 79.0. These results show that aloe-emodin was the most active compound against the three strains and that structurally different anthraquinones are a major source of new trichomonacidal compounds. In related studies, we have shown that some of the mentioned anthraquinones exhibited antioxidative and anti-inflammatory properties in chemical assays and macrophage cells [104], and that purpurin added to a high-fat diet significantly reduced weight gain in mice [105]. Could purpurin protect individuals against both trichomoniasis and obesity?

### Alkaloids - lycorine, lycosinine, and candimine

The Brazilian investigators Giordani and collaborators demonstrated in a series of studies the potential of extracts of the Amaryllidaceae plant family and their bioactive alkaloids to inhibit the growth of *T. vaginalis* as well as their anti-trichomonad mechanism of action. The following are highlights of some of their findings. The Amaryllidaceae plant family is known for its ornamental value and is also of medicinal interest owing to the occurrence of alkaloids with biological properties. Vieira, Giordani [23] evaluated the anti-*T*. *vaginalis* activity of eighteen methylene chloride (dichloromethane) extracts (12.5 to 0.19 mg/mL) and isolated six alkaloids (125 to 1.9 μg/mL) from the Amaryllidaceae species. The alkaloids diminished the viability of the trophozoite stage (from 15 to 40%). The extracts from *Hippeastrum breviflorum* demonstrated the highest anti-*T. vaginalis* activity (viability was reduced by 60%). Six fractions from a bioguided study with antiprotozoal activity had lycorine and lycosinine (Fig. 2) as major anti-parasitic components. The higher activity of the extracts when compared with activities of the isolated compounds suggests a synergistic effect of multiple compounds in the extracts. Vieira, Giordani [23] used the following procedure to obtain the extracts and isolated alkaloids. The crude extracts obtained after maceration of fresh bulbs in 96% ethanol at room temperature for 15 days were acidified with a solution of 10% HCl. The neutral material was then removed with petroleum ether, the solution was adjusted with 25% ammonium hydroxide to pH 9–10, and then extracted with dichloromethane and *n*-butanol. The alkaloids were then isolated by chromatography and their purity and structures confirmed by high-performance liquid chromatography (HPLC), nuclear magnetic resonance, and mass spectrometric analyses. The described methods offer a useful guide for future efforts designed to obtain pure bioactive compounds from plant sources. Giordani, Weizenmann [15] reported that the Amaryllidaceae alkaloids candimine and lycorine inhibited *T. vaginalis* nucleoside triphosphate diphosphohydrolase and ecto-5′-nucleotidase activities, possibly enhancing the susceptibility of the pathogen to host immune responses. The mechanisms of alkaloid-induced cell death seems to involve the arrest of the cell cycle by a pathway that does not involve criteria that are associated with apoptosis or apoptosis-like death [106].

To clarify the mechanism of cell death further, Giordani, Junior [107] evaluated the anti-trichomonad activity of lycorine and six aliphatic and aromatic esters at concentrations of 125 μM and 250 μM. About 60% of the parasites remained viable after exposure for 24 h to both concentrations of lycorine. By contrast, with the lauroyl ester, the observed reduction was to 16 and 3% residual activity at the two concentrations, respectively, suggesting that the lipophilicity of the fatty acid ester associated with the lauroyl side chain might facilitate its penetration through the amitochondriated layer of the parasite, resulting in enhanced cytotoxicity. The authors also suggest the data imply that: (a) the free (unprotected) OH groups at the C-1 and C-2 position of the lycorine molecule are not involved in the antiparasitic activity; and (b) esterification of lycorine with other lipophilic fatty acids at one or both OH groups has the potential to improve the inhibition of *T. vaginalis*. A related study found that besides having anti-*T. vaginalis* activity, lycorine modulates ectonucleotidases and stimulates neutrophils to secrete reactive oxygen species (ROS), suggesting that this mechanism of action exerted by the alkaloid could enhance the susceptibility of *T. vaginalis* to host immune cells, contributing to protozoan clearance [108].

These results demonstrate the antiprotozoal potential of the multiple bioactive compounds in the Amaryllidaceae plant species against *T. vaginalis*.

### Benzopyran HP1 and Uliginosin B

Cargnin, Vieira [11] found that uliginosin B and three benzopyrans (Fig. 2) isolated from *Hypericum polyanthenum*, a Brazilian native plant, had anti-*T-vaginalis* activity that was associated with damage to the cell membrane (> 90% release of lactic dehydrogenase, LDH). Benzopyran HP1 had the best activity against both susceptible and metronidazole-resistant clinical isolates (TV-LACM2), with no cytotoxicity towards mammalian cells. The authors suggest that benzopyran HP1 with significant antiprotozoal activity and selectivity seems to be a promising candidate for further study.

### Hedargenin

A review (meta-analysis) by Mehriardestani, Aliahmadi [19] that discusses reported anti-trichomonad effects of 13 medicinal plants and some of their bioactive purified compounds identified 95 relevant in vitro and clinical studies, including human studies. Alkaloids, isoflavonoid glycosides, essential oils, lipids, saponins, and sequiterpene lactones isolated from a number of medicinal plant families were shown to possess anti-trichomonad activity. These include cardimine, lycorine, methyl jasmonate and 12 other compounds with defined structures that exhibited ‘remarkable’ anti-trichomonad properties. The pentacyclic terpenoid hedargenin (Fig. 2) from the bark of *Cassnia holstii* had the best activity, with an IC_50_ value against *T. vaginalis* of 2.8 μM. The fact the structures of the alkaloids lycorine, lycosinine, berberine, and lirodenin contain the 1,3 benzodioxole ring system suggests that this functional group might be involved in the inhibitory mechanism and other reported bioactivities.

### Methyl jasmonate

Because mitochondria are target organelles of jasmonates, plant lipids that act as stress phytohormones that help protect plants against stress conditions such as drought and phytopathogens, Ofer, Gold [24] wanted to find out if methyl jasmonate (Fig. 2) would induce cell death in the amitochondriate *T. vaginalis* (lacking mitochondria). They found that cell death did occur following exposure of the parasite to methyl jasmonate and was associated with an apoptotic-like mechanism without the apparent DNA laddering sub G (1) cell cycle stage peak and caspase-3-activation, similar to the effect observed with metronidazole. Additional studies showed that methyl jasmonate was also cytotoxic to a metronidazole-resistant strain of *T. vaginalis* (ATCC 50143). The authors suggest that the mitochondria-independent cytotoxicity of methyl jasmonate could enable it to serve as a potential new agent against trichomoniasis. Vilela, Menna-Barreto [109] confirmed, using flow cytometry and scanning and transmission electron microscopy, that methyl jasmonate induced cell death and loss of hydrogenosomal membrane potential in *T. vaginalis*.

Gunjegaonkar and Shanmugarajan [110] note that the antioxidant and antimicrobial properties of methyl jasmonate are the basis for its use as a food preservative and that its anti-inflammatory properties provide a rationale for clinical applications. The above-mentioned results with *T. vaginalis* indicate this could include the treatment of trichomoniasis. Potentially other plant stress hormones and bioactive compounds, including auxins, gibberellins, cytokinins, abscisic acid, salicylic acid, brassinosteroids, and strigolactones that are synthesized within specialized plant cells, could exhibit anti-trichomonad properties.

### Betulinic and ursolic acids and derivatives

Innocente, de Brum Vieira [12] found that betulinic and ursolic acids (Fig. 2) and the piperazine derivative of betulinic acid inhibited the growth of *T. vaginalis*. The derivative showed greater activity than the parent compound. Vieira, Silva [111] found that *Manika rufula* plants from the Caatinga desert region of Brazil contained a number of characterized compounds that inhibited *T. vaginalis*. These included the α-amyrin caproate, β-amyrin caproate, lupeol acetate, and ursolic acid. After incubation for 2 h, ursolic acid reduced about 98% of parasite viability and induced drastic ultrastructural alterations in the cells, as observed by scanning electron microscopy. Bitencourt, de Brum Vieira [31] reported that the triterpene compound ursolic acid and its derivative, 3-oxime-urs-12-en-28-oic-ursolic acid, were active against fresh clinical isolates of *T. vaginalis* with an MIC of 25 μM. The derivative also inhibited a metronidazole-resistant *T. vaginalis* isolate and was not active in a hemolysis assay or cytotoxic to Vero cells, suggesting its value as potential trichomonocidal agent. Hübner, de Brum Vieira [112] also found that derivatives of betulinic acid inactivated *T. vaginalis*.

### Saponins

Rocha, de Brum Vieira [27] found that saponins (glycosylated triterpenes) isolated from *Quillaja, Passiflora,* and *Ilex* plant species had high anti-*T. vaginalis* activity with an MIC of 0.025%*.* All samples induced erythrocyte lysis and release of LDH. An evaluation by Damke, Tsuzuki [28] of purified saponins of the medicinal plant of *Sapindus saponaria*, used traditionally to cure ulcers, external wounds, and inflammation revealed their inhibitory potency against *T. vaginalis* with an MIC value of 0.156 mg/mL for nonclinical strains and 0.078 mg/mL for a clinical strain. Jain, Kumar [113] reported on a related study on the potential value of *Sapindus saponaria* trichomonocidal saponins for prophylactic contraception.

An uncommon saponin (**saponin 1**, Fig. 2) isolated by de Brum Vieira, Silva [114] from the Brazilian medicinal plant *Manilkara rufula* (Sapotaceae family) also known as ‘macarunduba’ inactivated *T*. *vaginalis* in vitro without toxicity against the human vaginal HMVII cells, suggesting that the compound does not affect host cells. Additional studies of mechanistic interest show that the compound reduced the cytoadherence of the pathogen to host cells, did not affect ROS production by neutrophils, did not hemolyse erythrocites, and disrupted the cell membrane of the pathogen. The authors suggest that the trichomonocidal effect against *T*. *vaginalis* is due to profound cell membrane damage.

Saponins are another class of natural compounds that merit further study for their anti-trichomonad properties in rodents and humans. de Groot and Müller-Goymann [50] confirmed that saponins such as digitonin and tomatine interact strongly with model membranes as well as natural membranes of erythrocytes, suggesting that their anti-trichomonad effect might involve disruption of the *T. vaginalis* cell membrane.

### Fungal compounds – pyrrolocin a

Patridge, Darnell [115] found that the bioactive 3-decalinoyltetramic acid (pyrrolocin A) (Fig. 2) they isolated from the Amazonian fungal endophyte E6927E inhibited the growth of the pathogenic bacteria *Staphylococcus aureus* and *Enterococcus faecalis* as well as isolates of the pathogenic fungal strains *Candidas albicans* and *Aspergillus* sp. King, Carter [25], using an image-based, high-throughput, and high-content for testing natural products for anti-trichomonal activity, discovered that pyrrolocin A was also a potent inhibitor of *T. vaginalis* with an effective inhibitory concentration (EC_50_) of 60 nM. Because the fungal compound also showed limited toxicity towards mammalian cervical cells with a high selectivity index of > 100, the results of the two investigations suggest that pyrrolocin A has the potential to protect food and humans concurrently against contamination and infection by pathogenic bacteria, fungi, and *T. vaginalis*. Will pyrrolocin A, with the exceptional high in vitro activity against *T. vaginalis*, also show similar activity after oral consumption or intravaginal application in women? Will pyrrolocin A-supplemented liquid and solid food protect humans against microbial, fungal, and protozoal infections? Will pyrrolocin A also be active against antibiotic-resistant bacteria, fungi [116, 117] and protozoa in vitro and in vivo? We are challenged to respond to these research needs. (Also see the above section on marine compounds for a description of anti-trichomonal effects of compounds from marine fungi).

### Photodynamic therapy

Two investigations describe photodynamic treatment as a potential new anti-trichomonad therapy. da Silva, Ribeiro Cde [118] found that exposure of *T. foetus* protozoa to light sensitized with an aluminum phthalocyanine tetrasulfonated photosensitizer efficiently killed the bovine pathogen. The associated morphological changes in the cells included membrane projections, nucleus fragmentation, endoplasmic reticulum proliferation, and cytoplasmic vacuolization, suggesting the possible value of the treatment for bovine trichomoniasis. Silva Fonseca, Alacoque [119] exposed metronidazole sensitive and resistant *T. vaginalis* strains to methylene blue as the light-emitting diode (LED). They found that light alone was ineffective, but the sensitized light inhibited up to 81% of the nonresistant and 91.1% of the resistant strains, respectively. The authors suggest that the effective photodynamic therapy offers great potential for routine use in women with trichomoniasis.

### Mechanisms of anti-trichomonad effects

In addition to mechanistic aspects mentioned above, here we briefly outline additional efforts that help clarify the anti-trichomonad mechanisms that are applicable to different classes of compounds.

Dirkx, Boyer [120] found that *T. vaginalis* has the gene-coding capacity to produce the enzyme beta-fructofuranosidase that is capable of hydrolyzing di- and tri-saccharides such as sucrose containing a terminal non-reducing fructose residue, suggesting that the inability of sucrose to support the growth of *T. vaginalis* is not due to the lack of an enzyme that can degrade (catabolize) the disaccharide. Puente-Rivera, Villalpando [121] characterized the previously identified 50 kDa metalloproteinase aminopeptidase P member TvMP50 as a new zinc-ion-mediated parasite virulence factor. These observations and additional studies suggest that the presence of TvMP50 during male trichomoniasis might explain the survival of *T. vaginalis* within the adverse conditions of the male urogenital microenvironment. In a follow-up study, Arreola, Villalpando [122] reported that the TvMP50 metalloproteinase is a monomeric aminopeptidase P-like enzyme. Will inhibition of the virulence enzyme protect males against trichomoniasis? O’Donoghue, Bibo-Verdugo [16] showed that clinically approved cancer drugs that inhibit the proteasome also have high activity in the enriched *T. vaginalis* in cell-free assays. The proteasome inhibitor carmaphycin-17 (Fig. 2) derived from the marine natural product carmaphycin showed greater activity against *T. vaginalis* than the reference drug metronidazole, as well as the ability to overcome metronidazole resistance. The authors suggest that these observations and additional mechanistic studies validate the proteasome of *T. vaginalis* as a therapeutic target for the development of a novel class of trichomonacidal agents. Quan, Kang [123] found that cervical epithelium cells exposed to *T. vaginalis* for 4 h produced intracellular and mitochondrial ROS in a parasite-load-dependent manner and that subsequent treatment with the ROS scavenger N-acetyl-L-cysteine [CH_2_(SH)-CH (NHCOCH_3_)-COOH, NAC] reversed the effect on apoptosis and NF-*κ*B inactivation of the cells. Another example is the observation by Sharma, Kumar [124] that a synthetic thiol compound eliminated *T. vaginalis* more efficiently than metronidazole via sulfhydryl-disulfide interchange reactions. The authors suggest that this represents a proof of concept for the selective targeting of Trichomonas. This proof of concept is supported by our observation that modification of disulfide bonds of the enzyme that inhibits trypsin in soy flour by cysteine [(CH_2_(SH)-CH (NH_2_)-COOH] and NAC through formation of mixed disulfides resulted in loss of inhibitory activity and in increased digestibility and nutritional value in rats [125]. Possible molecular mechanisms for these events might involve the initial reaction of the added sulfhydryl (thiol) compound (R-SH – cysteine, NAC, etc.) with a trichomonas protein disulfide bond (P-S-S-P) to form R-S-S-P (a mixed disulfide) + P-SH (disulfide-reduced protein). These transformations could produce biologically inactive trichomonad proteins with mixed and rearranged disulfide bonds [126] that might be responsible for the loss of pathogenicity. These observations imply that SH-antioxidants can protect cells against oxidative damage by *T. vaginalis*. Because antioxidative amino acid cysteine [(CH_2_(SH)-CH (NH_2_)-COOH] and the cysteine derivative NAC also protected frog embryos against adverse effects of acrylamide [127, 128], will cysteine, NAC, and the tripeptide glutathione, and other antioxidative (anti-free radical) sulfhydryl (SH)-containing compounds also protect human and animal tissues against adverse effects induced by ROS [129] produced by *T*. *vaginalis* and *T*. *foetus*?

The results from two related studies show major differences in the content and metabolism of sulfur and other amino acids in the two trichomonad species. Nozaki, Ali [130] reported that the metabolism of sulfur amino acids in *T*. *vaginalis* probably influences their virulence and defense against stress. Based on an extensive bioanalytical study, Westrop, Wang [131] showed that there are major differences in cysteine and methionine synthesis and metabolism in *T*. *vaginalis* and *T*. *foetus*, that both trichomonads synthesize methylthioadenosine by an unusual mechanism, that *T*. *foetus* has higher levels of ornithine and citrulline than *T*. *vaginalis* and released high levels of the polyamine putrescine, and that *T*. *vaginalis* but not *T. foetus* exported 2-hydroxyisocaproic acid derived from leucine. Because these biochemical events and associated enzymes probably contribute to the viability of the protozoa, inhibition of the active sites of the enzymes might transform pathogenic to nonpathogenic trichomonads.

On the basis of the observed inhibition of both *T*. *vaginalis* and *T*. *foetus* by synthetic diamine compounds and its reversal by the natural putrescine, Rigo, Trein [132] suggest that their mechanism of action might be via the polyamine pathway that is distinct from that observed with metronidazole. These results imply that diamines have the potential to reduce the in vivo pathogenicity of the two trichomonads.

A detailed study by Bradic, Warring [133] using high-throughput methods for large-scale genetic comparison of many strains of resistant and sensitive isolates of *T. vaginalis* was used to identify a panel of biomarkers associated with resistance that can be used as a diagnostic tool. Will these biomarkers help guide studies designed to overcome resistance?

The cited studies indicate the complexity of the anti-trichomonad mechanisms, where structurally different natural and synthetic compounds seem to act on metabolic pathways of the protozoa that are similar to or different from those observed with metronidazole. Which mechanistic inactivation pathway of anti-trichomonad compounds will be effective against both susceptible and metronidazole-resistant protozoa strains in vivo?

### Safety/toxicity aspects of anti-trichomonad compounds

We do not know if an anti-trichomonad compound that is effective in cell assays might also be effective in farm and domestic animals and in humans. The present study indicates that most of the anti-trichomonad compounds listed in Table 1 have not been evaluated for their efficacy in vivo. A major objective of the present review is to motivate clinicians and veterinarians to evaluate the therapeutic potential of the most active compounds listed in the table against trichomonad strains that infect humans and animals and described extracts containing multiple bioactive compounds. Such studies should include determination of the ratio the effective to toxic dose levels. The higher the ratio, the safer the compound.

Here, we will also briefly mention highlights on the safety of selected anti-trichomonads listed in Table 1. As mentioned earlier, the higher the value of the selectivity index (SI) of an anti-trichomonad in cell assays, the greater the safety. Cargnin, Vieira [11] reported a high SI for the anti-trichomonad benzopyran HP1 and a low one for uliginosin B. A summary of the widely studied safety of the anti-trichomonad potato glycoalkaloids α-chaconine and α-solanine by Elkahoui, Bartley [47] notes that because high levels of these compounds are reported to induce adverse effects in some cells as well as in vivo, it seems prudent to include safety assessment in any future in vivo studies. Kim, Nam [55] found no apparent adverse effects in mice consuming diets supplemented with the anti-trichomonad tomato glycoalkaloid tomatine. Some individuals consume high-tomatine pickled green tomatoes with no apparent adverse effects [134]. The anti-trichomonad caffeic and chlorogenic acids and quercetin from potatoes and resveratrol from grapes are widely consumed safe food ingredients. The use of probiotics in the treatment of trichomoniasis in women seems to reduce the adverse effects of metronidazole [95]. Finally, Zhao, Lu [135] reported that because the anti-trichomonad emodin prolonged the lifespan of the invertebrate animal *Caenorhabditis elegans*, this apparently safe compound might serve as an anti-aging dietary supplement.

## Conclusions

The worldwide use of herbal medicines is increasing, although without the apparent adequate education of physicians and consumers about their efficacy and safety [136]. It is therefore not surprising that plants and their extracts have become the focus of studies on their activity against pathogenic protozoa such as *T. vaginalis*. The results of the studies cited here describe the potential of food, marine, and medicinal plant extracts and isolated pure compounds to inhibit the growth of *T. vaginalis* parasitic strains that cause the sexually transmitted disease trichomoniasis in humans and the inhibition of the related *T. foetus* strains that infect farm and domestic animals. The studies indicate that some of the natural plant extracts and bioactive compounds are highly active against these parasites, suggesting that they might be able to fully or partially replace the widely used drug metronidazole, especially against resistant trichomonad strains.

Highly active formulations include potato and tomato glycoalkaloids, black tea extracts, and several essential oils and plant and marine extracts. There is also progress in the therapy to expose the protozoa to photosensitized light. It would be of interest to compare the therapeutic efficacy of treatment by light with some of the anti-trichomonad formulations derived from natural sources. This is, however, still a challenging problem because only a few studies have reported on medicinal plants that have a similar efficacy against *T. vaginalis* in infected women to that observed with metronidazole. One of the plants, *Zataria multiflora,* had a dual benefit: it inhibited both *T. vaginalis* and inactivated pathogenic bacteria in the vagina that cause vaginosis, which often accompanies trichomoniasis. There is, therefore, an urgent need to evaluate some of the other anti-trichomonad formulations based on effective natural and safe compounds and extracts in human trials for their efficacy against both trichomoniasis and vaginosis, as well as their efficacy in infected cows, bulls, pigs, cats, and dogs. Some of these compounds could be used in conjunction with probiotics to restore or modify the natural microbiota of the urogenital environment. We hope and anticipate that this review will stimulate interest in such studies, in view of the fact that several of described anti-trichomonad compounds have been reported to have additional health-promoting properties, including anti-oxidative, anti-inflammatory, anti-acrylamide, anti-obesity, anti-carcinogenic, anti-microbial, anti-viral, anti-malarial, and anti-aging effects. As noted above, the use of the described extracts and isolated pure compounds in the prevention and treatment of trichomoniasis and other diseases should be guided by the ratio of preventive/therapeutic to toxic dose. A combination of low doses of metronidazole with the active extracts and isolated compounds might act synergistically with reduced side effects to benefit human health.

Finally, the need for new treatments is strikingly illustrated by the report from the National Institute of Allergy and Infectious Diseases, National Institute of Health (NIH) [5] that notes the need for new therapeutics to help overcome the global epidemic of sexually transmitted infections, including trichomoniasis, chlamydia, gonorrhea, and syphilis. Will the anti-trichomonad compounds and extracts described in the present study also inhibit pathogens that cause chlamydia, gonorrhea, syphilis, the coronavirus and the human immunodeficiency (HIV) virus?

## Data Availability

Not applicable.

## Abbreviations

BV: Bacterial vaginosis
CSTs: Microbiota states or community types
EC_50_: Effective concentration that inactivates (inhibits, eliminates, kills) 50% of the cells under the test conditions
GC-M: Gas chromatography-mass spectrometry
HIV: Human immunodeficiency virus
IC_50_: Concentration that inhibits 50% of the cells under the test conditions
IL-6: Interleukin 6
LDH: Lactic dehydrogenase
LED: Light-emitting diode
HPLC-PAD: high-performance liquid chromatography with pulsed amperometric detection
MIC: Minimum inhibitory concentration
MLC: Minimum lethal concentration
ROS: Reactive oxygen species
STD: Sexually transmitted disease
TNF-α: Tumor necrosis factor-α
